# Chronic airflow obstruction in Tanzania – a cross-sectional study

**DOI:** 10.1186/s12890-018-0577-9

**Published:** 2018-01-19

**Authors:** Thomas Zoller, Elirehema H. Mfinanga, Tresphory B. Zumba, Peter J. Asilia, Edwin M. Mutabazi, David Wimmersberger, Florian Kurth, Francis Mhimbira, Frederick Haraka, Klaus Reither

**Affiliations:** 1 Swiss Tropical and Public Health Institute, Basel, Switzerland; 2 Ifakara Health Institute, Dar es Salaam, Tanzania; 3 Charité - Universitätsmedizin Berlin, corporate member of Freie Universität Berlin, Humboldt-Universität zu Berlin, and Berlin Institute of Health, Berlin, Germany; 4 Department of Infectious Diseases and Respiratory Medicine, Charité – Universitätsmedizin Berlin, Berlin, Germany; 5 University of Basel, Basel, Switzerland; 6 Clinical Research Unit, Schweizerisches Tropen- und Public Health Institut, Socinstr. 57, 4051 Basel, Switzerland

**Keywords:** COPD, Airflow obstruction, Tuberculosis, Tanzania

## Abstract

**Background:**

Chronic obstructive pulmonary disease is a global problem and available data from sub-Saharan Africa is very limited.

**Methods:**

A cross-sectional facility-based pilot study among patients and visitors to an urban and a rural primary healthcare facility was conducted in coastal Tanzania. The primary outcome was the prevalence of chronic airflow obstruction.

**Results:**

The final analysis included 598 participants with valid post-bronchodilator spirometry. Applying ATS/ERS spirometric criteria, chronic airflow obstruction was found in *n* = 24 (4%, CI_95_ 2.7–5.9) participants and in *n* = 30 (5%, CI_95_ 3.5–7.1) applying GOLD spirometric criteria. To analyse risk factors for chronic airflow obstruction including those not meeting ATS/ERS or GOLD criteria, FEF_25–75_ and FEV1% predicted was analysed in participants without evidence of pulmonary restriction among those exposed or not exposed to risk factors (*n* = 552). FEV1% predicted, but in particular FEF_25–75_ decreased with increasing symptom severity of shortness of breath as well as limitations in daily activities of participants. Cooking in general and cooking with biomass fuels vs. gas or electricity was associated with significantly lower FEF_25–75_, but not with lower FEV1% predicted. Participants having refrained from taking a job because of shortness of breath exhibited lower FEF_25–75_ (*p* < 0.01). A history of prior active TB was the most relevant risk factor associated with a decrease in FEF_25–75_ as well as FEV1% predicted.

**Conclusion:**

This study demonstrated a relevant prevalence of chronic airflow obstruction in primary healthcare attendants and healthy visitors of a Tanzanian hospital. Using the baseline data provided, larger and population-based studies are needed to validate these findings. TB may have more impact on development of chronic airway obstruction than smoking in Africa. Due to the influence of age on the GOLD definition of chronic airflow obstruction, studies should report results using both ATS/ERS and GOLD definitions and include age-stratified analysis. Analysis of FEV1 and in particular FEF_25–75_ may yield additional information on risk factors and earlier stages of chronic airflow obstruction.

## Background

Chronic airflow obstruction (CAO), caused by a chronic inflammation of airways in response to exposure to dusts and fumes is a characteristic feature of chronic obstructive pulmonary disease (COPD). CAO leads to a decline in lung function and to subsequent loss of exercise capacity. Cigarette smoking is the major causative factor for CAO in industrialized countries [1], leading to an overall population prevalence between 5% and 25% in Europe, North America, Latin America and Asia [2–5].

COPD is a disease mainly affecting higher age groups and most studies have so far addressed the population above the age of 40 years. Little is known however on prevalence and risk factors of COPD in developing countries with younger populations, and particularly in sub-Saharan Africa. Recently, the BOLD study investigated prevalences of CAO on a global level and provided data from Latin America and Asia as well as data from two study sites in Africa [2, 3, 6, 7]. Previous studies in sub-Saharan Africa have mostly focussed on risk groups with occupational exposure such as miners [8–11].

Epidemiology and risk factors of COPD in developing countries likely differ from those in the developed world. While cigarette smoking is less common in developing countries, factors like prolonged exposure to biomass fuels from cooking starting from early childhood, cooking indoors in rooms shared for living and sleeping purposes, high TB prevalence and different age structure have relevant influence on COPD epidemiology [12]. At present, it is unclear which effects this life-long exposure to biomass fuels in Africa has for the development of chronic obstructive pulmonary disease. Moreover, there is little data on chronic obstructive pulmonary disease in young African populations.

To gather data on CAO in Tanzania, we examined patients attending primary healthcare facilities as well as persons accompanying patients using a questionnaire and performed pre- and post-bronchodilator spirometry. Tanzanian Lung Health Study (TLHS) aims to provide first estimates on CAO in Tanzania and to explore risk factors as well as medical and social aspects of CAO, providing baseline data for further studies on a larger scale. Moreover, TLHS aims to give additional information on analysis of spirometric outcomes other than the standard definitions of CAO and their diagnostic value to detect earlier stages of small airways disease in an African setting.

## Methods

A cross-sectional facility-based study was conducted among attendants, accompanying persons and visitors to the rural Bagamoyo District Hospital (Bagamoyo, Pwani, Tanzania), and the urban Mwananyamala Regional Hospital (Dar es Salaam, Tanzania) between October 2015 and September 2016. Both study centres are located in coastal Tanzania without mining industry or exposure to other region-specific lung hazards. The primary outcome was spirometric evidence of CAO. Secondary outcomes were risk factors and parameters indicating social impact. Participants were eligible for inclusion if they were at least 18 years of age and either attendants of a primary healthcare clinic for any reason other than an acute respiratory problem, persons accompanying patients or visitors to the hospital visiting inpatients. Participants visiting the hospital for other reasons, having medical contraindications against bronchodilator drugs or having any other physical or mental condition limiting the ability to obtain a valid spirometry result were excluded (hospitalisation within <3 months, newly diagnosed heart disease within <3 months, uncontrolled hypertension, resting heart rate > 100/min., uncontrolled diabetes, pregnancy, acute infections or fever, acute pain, smoking during the last 2 h, thoracic or abdominal surgery within <3 months or any other severe physical or mental disease limiting the ability to comply with instructions for spirometry). To allow analysis of outcomes in patients across all relevant age groups, age-stratified sampling was used. Within age-strata, participants were invited randomly using a predefined selection procedure.

A sample size of 600 participants was intended to provide an acceptable prevalence estimate with 2.5% precision for this pilot study assuming a prevalence of 10% of chronic airflow obstruction.

After obtaining informed consent either in writing or by an impartial witness, participants were interviewed by a trained physician or a study nurse to obtain data on lung health and TB history and trained in performing spirometry supported by a video.

After spirometry was performed, patients inhaled 4 times 100 μg of salbutamol under supervision of a nurse using a spacer. Spirometry was repeated after 15 min. Quality criteria for spirometry were applied according to the 2005 ATS/ERS guidelines [13]. Spirometer readings were checked for acceptability and repeatability criteria by the physician performing the test and checked again by a senior pulmonologist. Only spirometer readings meeting all acceptability as well as repeatability criteria (minimum grade A and B) were finally accepted. Spirometry was performed using the NDD EasyONE World Spirometer (Zürich, Switzerland). Spirometric prediction equations of the Global Lung Function Initiative for African Americans were used [14].

Spirometric data was analysed for prevalence of CAO applying the FEV1/FVC < 5th percentile predicted (LLN) method (ATS/ERS spirometric criteria) as well as the GOLD definition with a fixed FEV1/FVC ratio < 0.7. A positive bronchodilator response was defined as an increase of FEV1 and/or FVC >12% and at least 200 ml compared to baseline. To further assess airflow obstruction including earlier stages, the post-bronchodilator forced expiratory flow at 25–75% of forced vital capacity (FEF_25–75_) [15] and the FEV1% predicted were used. As pulmonary restriction influences FEF_25–75_ and FEV1% predicted, participants with evidence of restriction (FVC < 5th percentile of FVC predicted and FEV1/FVC >0.85, as defined by ATS/ERS criteria [16]) were excluded from this analysis.

Data was analysed using JMP ver. 14. Median FEF_25–75_ and FEV1% predicted values were compared among participants exposed vs. unexposed to risk factors for CAO. Parameters were compared between risk groups using the Mann-Whitney-U-test to compare two and the Kruskal-Wallis-Test to compare multiple groups. Χ_**2**_ test was used to compare categorical outcomes between groups. Differences were considered significant when *p* was <0.05.

## Results

### Study population

A total of 1224 participants either attending or visiting the two health facilities of the study gave informed consent. 195 participants were excluded due to violating exclusion criteria and 32 participants due to incomplete datasets. 399 patients did not produce a post-bronchodilator spirometry meeting ATS/ERS quality criteria despite intensive video-assisted training and were excluded. The study population included 598 participants with valid post-bronchodilator spirometry.

We analysed if the population of participants not able to produce a spirometry according to ATS/ERS quality criteria was different from the study population in the final analysis. Slightly more women were excluded based on this criterion (54.6% vs. 45.3%, *p* = 0.05), but there was no significant difference in terms of age (median 46 vs. 45 years, *p* = 0.23), history of active TB infection (*p* = 0.93), visitors vs. patients (*p* = 0.4), education (*p* = 0.46), reason for seeking medical attention among patients (*p* = 0.71) and smoking status (*p* = 0.07).

### Demographic and social characteristics

The median age of the study population was 46 years (IQR 37–57 years) across the different age groups invited for participation, and 72% of patients were at least 40 years old (for details see Table 1). There was an equal gender distribution. The majority of participants (60%) were visitors to the hospital or persons accompanying patients to primary healthcare clinics. The level of education was generally low, with most participants having either no (29%) or only primary school (48%) education. Main occupations were business and trade (29%) as well as farming and agriculture (24%). The leading reasons for seeking medical attention for patients attending primary healthcare clinics were infections/fevers (17%), musculosceletal problems and injuries (15%) and cardiovascular problems (14%). The majority of participants cooked with charcoal (60%) and wood (25%), and only few participants with gas (8%).

**Table 1:** Demographic and social characteristics of the study population

		N	% of study population
Age group	18–39	169	28
	40–59	299	50
	60–79	124	21
	> = 80	6	1
Gender	Male	310	52
	Female	288	48
Type of participant	Patient of hospital	242	40
	Visitor to hospital	356	60
Education	No formal education	173	29
	Primary school	289	48
	Secondary school	99	17
	University/college	31	1
Occupation^a^	Business and trade	172	29
	Farming and agriculture	141	24
	Craftsmen	38	6
	Drivers	21	4
Reasons for seeking medical attention (patients)^a^	Infections/fevers	41	17
	Musculosceletal problems	35	15
	Cardiovascular problems	33	14
	Gastrointestinal problems	29	12
	Non-acute respiratory problems	25	10
	Urogenital/reproductive problems	13	5
Cooking fuel	Charcoal	360	60
	Wood in open stove	137	23
	Wood in open fire	12	2
	Gas	49	8
Smoking status	Never smoker	435	73
	Current or recent smoker	87	15
	Ex-smoker (≥6 months abstinence)	76	13

^a^Only major categories shown

### CAO prevalence and bronchodilator response

Applying ATS/ERS criteria, CAO was found in *n* = 24 (4.0%, CI_95_ 2.7–5.9) participants and *n* = 30 (5.0%, CI_95_ 3.5–7.1) met GOLD spirometric criteria for CAO. There was an obvious difference in CAO prevalence across age groups using the two definitions. Applying ATS/ERS criteria, prevalence of CAO increased only moderately with age from 3.6% to 5.6% in 4 age groups, whereas prevalence defined by GOLD spirometric criteria was only 1.8% in participants <40 years of age, but 11.3% in participants >60 years of age (for details see Table 2). Overall, *n* = 46 (7.7%, CI_95_ 5.8–10.1) showed a positive bronchodilator response. Most participants meeting either ATS/ERS or GOLD spirometric criteria had either mild or moderate levels of obstruction (see Table 3).

**Table 2:** Prevalence of chronic airflow obstruction in the study population according to spirometric criteria of ATS/ERS using FEV1/FVC < 5th percentile predicted (LLN) method or the Global Initiative for Chronic Obstructive Lung Disease (GOLD) criteria

Risk factor	Group	n	Meet criteria	%	CI_95_
CAO (LLN)	All	598	24	4.0	2.7–5.9
	Age groups				
	18–39	169	6	3.6	1.6–7.5
	40–59	299	11	3.7	2.0–6.5
	60–79	124	7	5.6	2.8–11.2
	> = 80	6	0	0	
CAO (GOLD)	All	598	30	5.0	3.5–7.1
	Age groups				
	18–39	169	3	1.8	0.6–5
	40–59	299	13	4.3	2.5–7.3
	60–79	124	14	11.3	6.8–18
	> = 80	6	0	0

**Table 3:** Severity of CAO according to spirometric criteria of ATS/ERS using FEV1/FVC < 5th percentile predicted (LLN) method or the Global Initiative for Chronic Obstructive Pulmonary Disease (GOLD) criteria

		n	%	FEV1% of predicted
CAO (LLN)	All	24		
	Very severe	2	8.3	<35%
	Severe	0	0	35–49%
	Moderately severe	3	12.5	50–59%
	Moderate	6	25.0	60–69%
	Mild	13	54.2	≥70%
CAO (GOLD)	All	30		
	Very severe	2	6.7	<30
	Severe	0	0	30–49
	Moderate	15	50.0	50–79
	Mild	13	43.3	≥ 80

### Correlation of symptoms and limitations of daily activities with spirometry

**Fig. 1:**
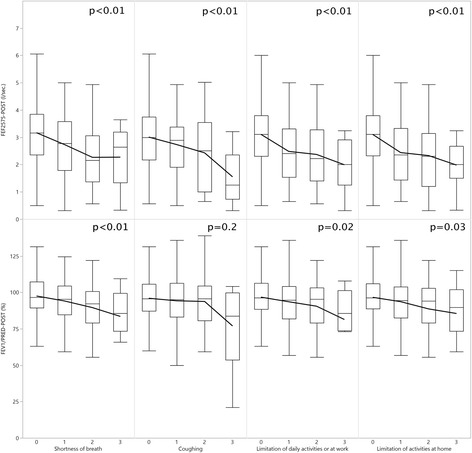
Chronic airflow obstruction in relation to severity of symptoms: post-bronchodilator median FEF_25–75_ (l/s.) and FEV1 in patients without restriction by symptom severity (0 no symptoms, 3 severe symptoms). Boxplot shows median & interquartile ranges and whiskers denote the furthest point within 1.5× interquartile range. Number of subjects in severity groups (from 0 to 3): shortness of breath *n* = 331, 140, 67, 13; coughing *n* = 447, 75, 20, 9; limitation of daily activities or at work *n* = 409, 84, 43, 15; limitation of activities at home *n* = 418, 81, 38, 14, respectively. Note: the symptom “coughing” was assessed in 4 severity grades. Only one participant graded coughing with severity 4; this participant and was removed from the figure, as no boxplot could be drawn from a single value. The patient had a FEF_25–75_ of 1.99 l/s and a FEV1/PRED of 76.39%

Current definitions of CAO only allow to define presence or absence of obstruction and may not capture more subtle changes in airflow obstruction; we analysed therefore FEF_25–75_ as well as FEV1% predicted values in participants without evidence of restriction (*n* = 552) in an exploratory analysis and correlated spirometric values with symptoms and limitations in daily life, and with parameters causing social impact. Participants graded the level of respiratory symptoms (shortness of breath, coughing) and social impact (limitation of daily activities or at work, limitation of activities at home) on a scale between 0 (no symptoms) and 3 (severe/frequent symptoms), and for coughing up to 4 (constant symptoms). The results are shown in Fig. 1. Symptom severity increased and indicators for social impact deteriorated with decreasing FEF_25–75_ and FEV1% predicted.

### Risk factors for airflow obstruction

To identify risk factors promoting airflow obstruction, data from the patient interview was used to classify patients into exposed and non-exposed groups and FEF_25–75_ as well as FEV1% predicted values in patients without restriction were compared. The results are given in Table 4. There was no significant difference in lung function parameters between participants who smoked or were exposed to dusts and fumes at their workplace. Cooking in general and cooking with biomass fuels vs. gas or electricity was associated with significantly lower FEF_25–75_, but not with lower FEV1% predicted. The biggest and significant differences in both lung function parameters were observed between participants with a history of TB vs. those who reported no history of prior TB (*p* < 0.01). Symptoms of chronic bronchitis were associated with a decrease in both lung function parameters (*p* = 0.02). Participants having refrained from taking a job because of shortness of breath exhibited lower FEF_25–75_ and FEV1% predicted values, but the difference was only significant for FEF_25–75_.

**Table 4:** Post-bronchodilator FEF_25–75_ (l/s.) and FEV1% predicted in participants without restriction, with risk factors, symptoms and limitations caused by chronic airflow obstruction in participants without evidence of restriction)

	Exposure	N	% of n	Median FEF_25–75_ (IQR), post bronchodilator (L/s.)	Δ FEF_25–75_ (L/s.)	*p*	Median FEV1% PRED (IQR)	Δ FEV1%PRED	*p*
Smoking	Current or former smoker	155	28.1	3.1 (2.15–3.76)	0.2	0.4	96.44 (87.39–104.12)	1.23	0.59
	Never smoker	397	71.9	2.9 (2.05–3.65)			95.21 (86.14–106.34)		
Exposure to dusts and fumes at workplace	Exposure	442	80.1	2.97 (2.03–3.7)	0.19	0.78	95.15 (85.94–104.42)	3.74	0.07
	No exposure	110	19.9	2.78 (2.2–3.63)			98.88 (89.31–107.82)		
Cooking	Regular cooking	317	57.4	2.74 (1.93–3.47)	0.43	<0.01	95.19 (85.61–105.79)	0.2	0.12
	No regular cooking	231	41.8	3.16 (2.34–3.99)			95.39 (88.62–105.37)		
Biomass fuels for cooking	Cooking with wood or coal	472	85.5	2.84 (1.96–3.61)	0.53	<0.01	95.15 (85.62–104.93)	1.33	0.39
	Cooking with gas or electricity	47	8.5	3.36 (2.5–3.99)			96.47 (72.93–106.29)		
History of active TB	History of active TB	71	12.9	2.07 (1.5–3.12)	0.95	<0.01	96.2 (87.48–106.34)	6.34	<0.01
	No history of active TB	481	87.1	3.02 (2.23–3.71)			89.86 (77.11–101.86)		
Chronic bronchitis (chronic cough and sputum)	Yes	46	8.3	2.41 (1.44–3.49)	0.58	0.02	90.73 (74.02–103.34)	4.86	0.02
	No	506	91.7	2.99 (2.1–3.67)			95.6 (86.89–105.86)		
Having ever refrained from taking a job because of shortness of breath	Yes	87	15.8	2.43 (1.56–3.28)	0.6	<0.01	95.25 (81.58–103.25)	0.28	0.14
	No	465	84.2	3.02 (2.17–3.71)			95.53 (86.99–105.96)		

## Discussion

Chronic airflow obstruction is an important cause of morbidity and mortality, and has major impact on the lives of those affected. Data from this pilot study in coastal Tanzania indicate a relevant level of CAO among urban and rural inhabitants. CAO can be found across all age groups and those affected may suffer from limitations in their ability to work and in quality of life.

This study showed that approximately one out of twenty inhabitants of coastal Tanzania has CAO according to both definitions currently used. Whereas the ATS/ERS definition using the LLN method showed only a moderate increase of prevalence with age, the GOLD definition showed a six-fold higher prevalence of CAO in the highest vs. the youngest age group. As the GOLD definition uses a fixed FEV1/FVC ratio of 0.7 as cut-off to define CAO, older age groups are overrepresented as the FEV1/FVC ratio declines physiologically with increasing age [17]. There is a persisting controversy on which classification to use; the LLN method indicating CAO is better suited for research purposes as it controls for ageing of the lung whereas the GOLD spirometric criteria are preferred by many clinicians due to their simplicity. The results from this study however underscore the concerns regarding over-diagnosis of elderly patients using the GOLD definition as well as potential under-diagnosis in younger patients, and demonstrate this effect in an African setting. Future epidemiological studies in Africa should therefore report results using both definitions until a universally accepted method has been developed. Moreover, results should include prevalences stratified by age groups, as exposure to biomass fuel smoke starting from birth or even in utero [12] may lead to an earlier onset and higher prevalence of CAO in young African populations.

Few studies have reported prevalence of CAO in general populations from sub-Saharan Africa not exposed to special risk factors (e.g. gold miners). The reports available so far do not give consistent results across African regions; the prevalences found in this study were lower than in a recent study from Nigeria (5.1%–10% (LLN)) [6], Malawi (14% (GOLD)) [18] or from Uganda (12.4% (LLN), 16.2% (GOLD)) [19], but higher than in Cameroon (2.4% (LLN), 0.5% (GOLD)) [20]. Consistent across all studies, the majority of patients had either mild or moderate severity of obstruction. There is still no sufficient data from large population-level spirometry-based and quality-assured studies in sub-Saharan Africa to give a good CAO prevalence estimate in general or specific to African regions; the data presented in this study add relevant baseline data for planning studies on a larger scale and underscore the importance of reporting data in different age groups.

Risk factors for CAO likely differ from other regions of the world due to different living circumstances, living standards and lifestyle of populations. Smoking [1] and exposure to particulate matter [21] in the air are the predominant risk factors for CAO in industrialized countries. Data from China, Latin America and Turkey show a moderately increased risk of CAO after exposure to smoke from biomass fuels, with a stronger effect for firewood than for charcoal [22]. Following these principles, indoor air pollution is generally assumed to be a major risk factor in Sub-Saharan Africa [23]. Four recent studies however did not demonstrate a clear association between indoor use of biomass fuel with CAO [6, 7, 19, 20]. Chronic airflow obstruction may begin early in life and investigation of first signs may be missed by applying the ATS/ERS or GOLD criteria only – the predominant method of analysis used in studies from Africa. Large population based epidemiological studies have however demonstrated excellent sensitivity for early detection of airway pathology using continuous analysis of FEV1 and in particular FEF_25–75_ [21, 24, 25] as well as FEV1 as a percentage of FVC [21]. In an exploratory approach, we therefore used FEF_25–75_ as well as FEV1% predicted to detect more subtle changes in airway pathology. Analysis of FEF_25–75_ seemed to have a higher sensitivity and better correlation with severity of symptoms and physical limitations than FEV1% predicted. Both parameters indicated differences among participants exposed and unexposed to risk factors for CAO typical for an African setting and indicated their potential significance for causing social impact. We therefore recommend that in larger studies on CAO in Africa, results should be reported both using the standard CAO definitions, but also using analysis of continuous spirometric values such as FEF_25–75_ and FEV1% predicted.

Recent meta-analyses and studies on a global scale were able to demonstrate that active tuberculosis as an infection causing chronic pulmonary inflammation over a prolonged period of time is a major factor contributing to CAO [26–28]. In this study, a history of active TB infection was the most relevant risk factor associated with a lower FEF_25–75_ as well as a lower FEV1% predicted. The observed effect of TB infection is consistent with other recently published studies from local African populations [6, 18, 29] and underscores the significance of TB for development of chronic lung disease in Africa.

This study had limitations. In order to examine CAO in a sufficient number of subjects in each age class in this study, age-stratified sampling was used. The population sample examined consisted of a mixed population composed of hospital visitors and patients. Therefore, particularly the prevalence of CAO should not necessarily be regarded as representative for the general population. An unexpectedly high number of study participants was unable to produce a spirometry meeting ATS/ERS quality criteria despite intensive and standardized pre-spirometry training including a training video two times. In previous studies in developing country settings in Latin America and Africa [6, 19, 30], observed spirometry failure rates were lower except for one study from Malawi with a similar rate of exclusions [7]. Local cultural and religious factors in this community causing people not to expose themselves in a medical examination requiring their participation with maximum muscular force used, together with a generally low level of education probably have contributed to this high failure rate. Main findings in this study, particularly with regard to risk factors for CAO need to be confirmed in larger population-based studies.

## Conclusions

In conclusion, this study demonstrated a relevant burden of CAO in a Tanzanian population and showed major differences in prevalence as well as age-specific prevalence between the two standard definitions of CAO used. Although most individuals with evidence of CAO had only mild or moderate obstruction, CAO likely has relevant social and social and economic impact for those affected. TB may have more impact on development of chronic airway obstruction than smoking in Africa. Analysis of FEV1 and FEF_25–75_ in epidemiologic studies on CAO may give important information on risk factors and impact in addition to using standard definitions for CAO alone.

## Abbreviations

ATS/ERS: American Thoracic Society/European Respiratory Society
BOLD: Burden of Obstructive Lung Disease Initiative
CAO: Chronic airflow obstruction
COPD: Chronic obstructive pulmonary disease
FEF: Forced exspiratory flow
FEV1: Forced expiratory volume in 1 s
FVC: Forced vital capacity
GOLD: Global Initiative for Chronic Obstructive Lung Disease
LLN: Lower limit of normal
TB: Tuberculosis
